# Type a botulinum toxin in the management of spontaneous salivary otorrhea: a case report

**DOI:** 10.1186/s40463-020-00457-y

**Published:** 2020-08-12

**Authors:** Teffran J. Chan, Trevor A. Lewis, Doron D. Sommer

**Affiliations:** 1 Michael G. DeGroote School of Medicine, McMaster University, Hamilton, Ontario Canada; 2 Division of Otolaryngology – Head and Neck Surgery, Department of Surgery, McMaster University, Hamilton, Ontario Canada

**Keywords:** Salivary otorrhea, Type a botulinum toxin, Management, Parotid, Injection

## Abstract

**Background:**

Spontaneous salivary otorrhea is a rare presentation only previously documented twice in literature where parotid salivary secretions are found in the external auditory canal. Conventional treatment of spontaneous parotid salivary fistulas includes surgical management with interposed grafts, fistula tract ablation, and possible superficial parotidectomy. Associated risks include facial nerve injury, Frey syndrome and facial scarring. Here we report the first case of spontaneous salivary otorrhea conservatively managed with a type A botulinum toxin (BTA) injection.

**Case presentation:**

A 17-year-old female presented with a 5-month history of left sided otorrhea and transient left facial swelling associated with gustatory stimulation. The otorrhea fluid tested positive for salivary amylase and negative for beta 2 transferrin. Fifty units of BTA were injected into the left parotid gland under ultrasound guidance. Cessation of symptoms was achieved 3 weeks after intervention. The patient remains symptom-free at the 2 year follow up.

**Conclusion:**

BTA injection was well tolerated under ultrasound guidance and has led to long-term resolution of the patient’s symptoms. BTA injection appears to be a safe and effective way to conservatively manage this rare presentation of spontaneous salivary otorrhea.

## Background

Salivary otorrhea is a rare condition that has been reported in congenital parotid fistulas, branchial cleft anomalies, and anatomical defects of the external auditory canal. However, to our knowledge, a spontaneous presentation has only been reported twice in literature [1, 2]. Clinical presentation includes production of clear, thin otorrhea in response to gustatory stimuli. Diagnosis has been confirmed with salivary amylase testing of discharge and sialography. Conventional surgical management include fistula tract excision with subsequent placement of temporalis fascia or tragal perichondrium to separate parotid gland from external auditory canal [2]. Depending on the etiology of the lesion, a superficial parotidectomy may be required. Here we present a case of a 17-year-old girl with unilateral spontaneous salivary otorrhea treated with type A botulinum toxin (BTA).

## Case presentation

A 17-year-old female was referred to the pediatric otolaryngology outpatient clinic after experiencing acute occipital pain, left sided otorrhea and swelling of the external auditory canal. The otorrhea was noted to continue for several weeks and her symptoms were exacerbated by gustatory stimuli or when undergoing dental procedures. In addition, she developed left facial swelling and intermittent headaches. Her past medical history was significant for Celiac disease and erythema nodosum. At the time of presentation to our clinic, the patient was concurrently being investigated for Behçet disease.

On physical examination, left facial swelling and clear, watery ear discharge was observed. No neck masses or sinus tracts were seen upon inspection. Under a microscopic examination, the tympanic membrane showed no retraction, cholesteatoma, or middle ear effusion. The external auditory canal had a normal contour with no mass or ectopic tissue.

Previous parotid gland scintigraphy with Technetium 99 m uptake, CT Head, CT Temporal bone, and neck ultrasounds performed at other centres revealed no anatomic abnormalities, such as branchial arch remnants or masses. MRI sialography showed normal salivary ducts and no abnormal extravagation of contrast. Beta 2 transferrin testing was negative, ruling out potential cerebral spinal fluid leak. However, the otorrhea tested positive for amylase (> 16,000 units/L) confirming a salivary etiology. A gram stain previously yielded gram-negative rods, gram-negative and positive cocci.

Treatment options were reviewed and the patient elected to proceed with a trial of BTA. Under sterile conditions, the patient’s superficial and deep lobes of the left parotid gland were injected with a total of 50 units of Botox® under ultrasound guidance using a 22-gauge spinal needle. There were no complications.

In post-procedural follow up, the patient reported diminishing otorrhea for 2–3 weeks. Otorrhea ceased after the third week; however, transient intermittent facial swelling continued to be reported with each episode lasting 1–2 h. These episodes gradually subsided and resolved 3 months after injection. Palpation at 3 months yielded minimal salivary secretion from the left Stenson’s duct and no otorrhea. At the 2 year follow up clinic visit, the patient reported no recurrence of any symptoms.

## Discussion

Spontaneous salivary otorrhea is a rare condition typically managed with surgical intervention. We report the successful use of BTA as a conservative therapeutic modality for spontaneous salivary otorrhea.

Type A botulinum toxin is a potent neurotoxin created by *Clostridium botulinum,* widely used for its paralytic effect at the neuromuscular junctions by inhibiting cholinergic signal transduction across the synapse. This is achieved by cleavage of SNAP-25, a component of the soluble n-ethylmaleimide – sensitive factor associated protein receptor (SNARE) complex, on the presynaptic nerves preventing acetylcholine release at the neuromuscular junction, thereby paralyzing the muscle [3]. This property has both cosmetic and therapeutic applications. In the context of salivation, BTA diminishes salivary excretion upon stimulation. This is achieved by BTA toxin disruption of the parasympathetic secretomotor pathway at the cholinergic nerve terminals [4, 5].

BTA injection in the parotid gland for sialorrhea was first cited by Bushara (1997) and has since been widely used in management of sialorrhea [6–9]. A retrospective study by Send et al., found BTA glandular injections had a 100% treatment success rate in patients with post-operative parotid sialocutaneous fistulas without any recorded adverse events [10]. Longitudinal studies has shown its use to be safe and well-tolerated for long term clinical use [11–13].

**Fig. 1:**
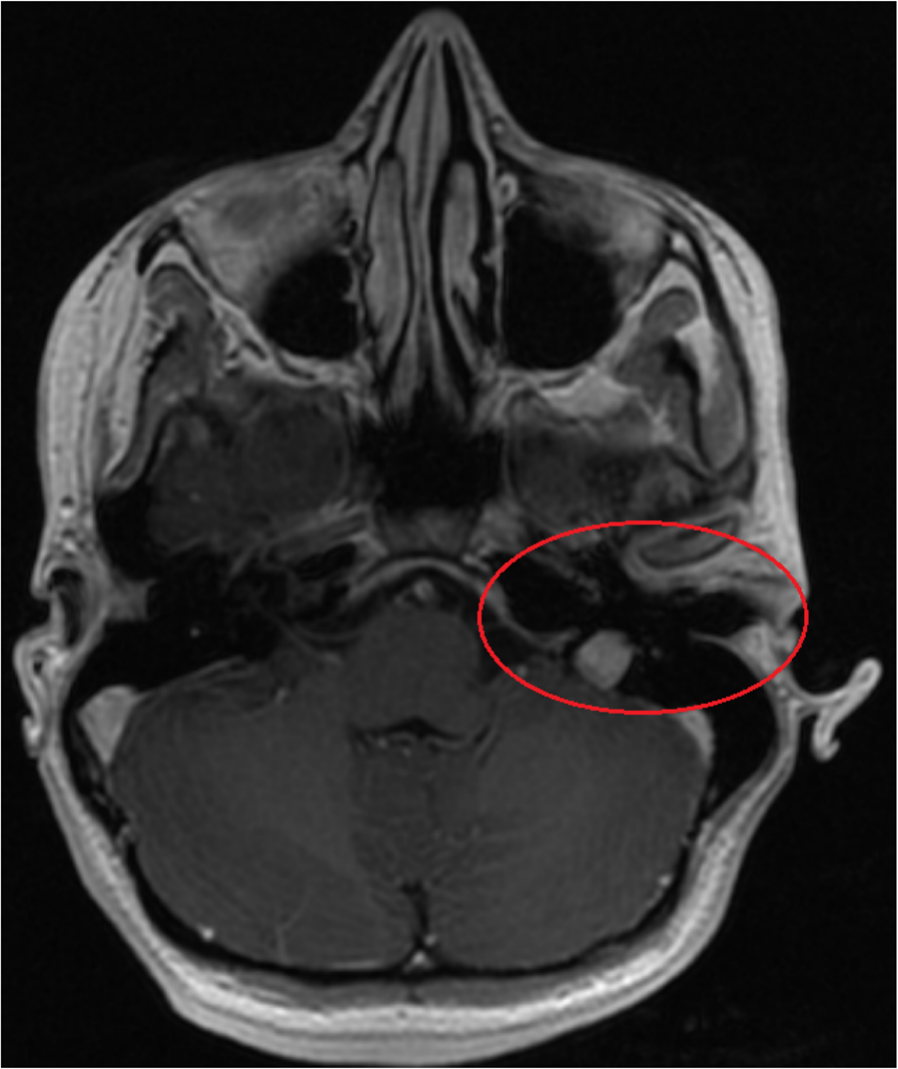
T1 contrast enhanced sequence with fat saturation a normal parotid and external auditory canal

The pathophysiology of the patient’s acute onset of salivary otorrhea is unknown at this point. However, with the clinical presentation and complex past medical history of autoimmune disorders, the cutaneous communication was hypothesized to be formed secondary to an inflammatory process. The diagnosis of salivary otorrhea was challenging due to inability to visualize the fistulous tract despite utilizing an array of diagnostic imaging techniques, including MRI sialography (Fig. 1). A similar encounter was described by Rana et al. where no tracts were visualized with multiple imaging modalities, but upon surgical exploration a soft tissue tract was appreciated [2]. We postulate this tract is only patent with mechanical pressure upon salivation, which would impede spontaneous tissue closure. Thus, utilization of BTA to stop salivary outflow from the parotid gland for 3-month period would allow spontaneous closure of the fistulous tract.

BTA injections into glandular tissue can be performed either under ultrasound guidance, or by palpation by experienced physicians [14]. Injection techniques are less invasive, costly and require less technical skill to perform compared to alternative surgical interventions. With superficial parotidectomies frequently indicated in treatment of salivary aural fistulas, facial nerve complications remain a significant concern. In a systematic review on surgical outcomes of 1317 patients undergoing superficial parotidectomies for benign parotid gland tumors, the incidence of facial nerve paresis and paralysis were 6.75 and 0.8% respectively [15]. In contrast, a longitudinal study on 65 patients receiving at least 3 injections of botulinum toxin A or B for sialorrhea reported no cases of long term facial nerve paralysis or paresis [12]. Nevertheless, mild to moderate transient side effects were noted by some patients in the study, including xerostomia, dysphagia, and viscous saliva [12, 14].

Limitations to BTA injection as the primary treatment is the associated cost. The cost of one unit of Botox® is $5.55 CAD ($277.35 per 50 IU) [16]. The cost of one unit of Xeomin® is $6.12 CAD ($302.00 per 50 IU) [10]. The cost of one unit of Dysport® is $4.69 CAD ($234.50 per 50 IU) [10]. However, cost variations exist depending on country of purchase and quantity of order [16]. Ultimately, cost of treatment will increase incrementally with administered units required per patient. No standardized dosing or treatment guideline has been established. However, an international consensus statement was published by Reddihough et al., in 2010 with recommendations of 10–50 U of BOTOX® per side or 15–75 U of Dysport® per parotid gland for patients with sialorrhea [17]. Case reports have shown neutralizing antibody formation in response to repetitive injections surpassing the lowest effective dose which can cause resistance to the clinical effects of BTA [3]. It must be noted, however, that the potency, clinical efficacy, and duration of effect are not uniform between serotypes, occasionally making dose-response assumptions unpredictable.

## Conclusion

We present the third known case of spontaneous unilateral salivary otorrhea, which was successfully treated with a BTA injection. Cessation of otorrhea was achieved three weeks after treatment without recurrence at the 2-year post-procedure time point. More cases are required to assess the efficacy of BTA injections as a definitive treatment, however, BTA appears to be a safe and well-tolerated conservative treatment for patients with salivary otorrhea. This therapeutic modality should be considered as a conservative management option for such conditions.

## Data Availability

Data sharing is not applicable to this study as no data sets were generated or analyzed during the current study. The report can be substantiated with patient documentation from clinical encounters.

## Abbreviations

BTA: Type A Botulinum Toxin
